# RBBP4 dysfunction reshapes the genomic landscape of H3K27 methylation and acetylation and disrupts gene expression

**DOI:** 10.1093/g3journal/jkac082

**Published:** 2022-04-13

**Authors:** Weipeng Mu, Noel S Murcia, Keriayn N Smith, Debashish U Menon, Della Yee, Terry Magnuson

**Affiliations:** Department of Genetics, Lineberger Comprehensive Cancer Center, The University of North Carolina at Chapel Hill, Chapel Hill, NC 27599-7264, USA

**Keywords:** histone methylation, histone acetylation, transcriptional regulation, Polycomb-group proteins, enhancer, super-enhancer

## Abstract

RBBP4 is a subunit of the chromatin remodeling complexes known as Polycomb repressive complex 2 and histone deacetylase 1/2-containing complexes. These complexes are responsible for histone H3 lysine 27 methylation and deacetylation, respectively. How RBBP4 modulates the functions of these complexes remains largely unknown. We generated viable *Rbbp4* mutant alleles in mouse embryonic stem cell lines by CRISPR-Cas9. The mutations disrupted Polycomb repressive complex 2 assembly and H3K27me3 establishment on target chromatin and altered histone H3 lysine 27 acetylation genome wide. Moreover, *Rbbp4* mutant cells underwent dramatic changes in transcriptional profiles closely tied to the deregulation of H3K27ac. The alteration of H3K27ac due to RBBP4 dysfunction occurred on numerous cis-regulatory elements, especially putative enhancers. These data suggest that RBBP4 plays a central role in regulating histone H3 lysine 27 methylation and acetylation to modulate gene expression.

## Introduction

Histone modifications are critical epigenetic regulators that specify the transcriptome during lineage determination and tightly control transcriptional states to preserve cellular identity over cell generations (Laugesen *et al.* 2019). Various histone modifications exist, including methylation and acetylation, which are central regulators of gene expression. These modifications are functionally deterministic on chromatin regions around cis-regulatory elements such as promoters, enhancers, and silencers. They impact transcription factor binding and activity by altering chromatin structure and accessibility (Creyghton *et al.* 2010; Ferrari *et al.* 2014). Lysine 27 on histone H3 (H3K27) can be either methylated by Polycomb repressive complex 2 (PRC2) (Cao *et al.* 2002) or acetylated by p300 and CBP (Jin *et al.* 2011). These modifications serve as hallmarks of transcriptional repression and activation, respectively. Disruption of H3K27 methylation and acetylation results in numerous cellular defects. These include cell proliferation and differentiation anomalies during embryogenesis and tissue specification (Faust *et al.* 1998; O’Carroll *et al.* 2001; Mu *et al.* 2014). Their dysregulation impacts disease processes such as tumorigenesis (Laugesen and Helin 2014).

PRC2, the methyltransferase with specific activity toward H3K27, is responsible for the mono-, di-, and tri-methylation modifications of this residue (H3K27me1/me2/me3) (Cao *et al.* 2002). PRC2 maintains gene silencing primarily through the deposition of H3K27me3 at promoter regions (Tanay *et al.* 2007; Ku *et al.* 2008). This activity has significant developmental implications. RBBP4 and RBBP7 are core subunits of PRC2 that share 92% identity. Both proteins contain WD40 domains that serve as scaffolds for protein complex assembly or platforms to recruit diverse molecules that form functional complexes (Verreault *et al.* 1996; Smith *et al.* 1999). Within PRC2, RBBP4 physically interacts with SUZ12 and AEBP2 (Chen *et al.* 2018). However, unlike EED, SUZ12, and EZH2, the RBBP4/7 homologs are not required for methyltransferase activity (Huang *et al.* 2021, p. 4), and precise roles for RBBP4/7 in regulating PRC2 functions remain unclear.

RBBP4/7 are also components of histone deacetylase (HDAC) complexes essential for normal development, including NuRD, Sin3, and CoREST (Kelly and Cowley 2013). RBBP4/7 and HDAC1 and HDAC2 are shared among these complexes and coordinate with other complex-specific subunits to provide target specificity or additional catalytic activities (Laugesen and Helin 2014). HDAC-complexes function in both transcriptional activation and repression through dynamic acetylation and deacetylation (Clayton *et al.* 2006; Kelly and Cowley 2013). The NuRD complex, comprised dimer of the subcomponents HDAC1:RBBP4:MTA1, binds nucleosomes, with the RBBP4 protein mediating interaction with histone H3 tails as a mechanism for recruitment of NuRD to chromatin (Millard *et al.* 2016). Histone acetylation and deacetylation regulation in transcription need clarification to understand how RBBP4 functions within different HDAC complexes.

Since RBBP4/7 are essential for cell viability, it is difficult to test their roles experimentally. By utilizing CRISPR-Cas9 to target *Rbbp4* and *Rbbp7* in mouse embryonic stem cells (ESCs), we generated viable ESC colonies with mutated *Rbbp4/7*. We found that RBBP4 mutations remarkably altered transcriptional profiles and the landscape of H3K27 methylation and acetylation. Our results suggest that RBBP4 is involved in acetylation and deacetylation of H3K27, especially at enhancers, to fine-tune gene activity.

## Materials and methods

### Cell culture

Mouse E14 ESCs were cultured in Glasgow Minimum Essential Medium supplemented with 15% fetal bovine serum, 1.0 mM l-glutamine, 0.1 mM minimal essential medium-nonessential amino acids, 0.1 mM β-mercaptoethanol, and leukemia inhibitory factor.

### Genome editing of *Rbbp4* and *Rbbp7* by CRISPR-Cas9

sgRNAs targeting *Rbbp4* and *Rbbp7* were cloned into eSpCas9(1.1) (Addgene, Cat. No. 71814) using a Golden Gate assembly cloning strategy (Bauer *et al.* 2014). *Rbbp4* sgRNA sequences are 5′-CACCGATCATTAGGGAGCTGGACAC-3′ and 5′-AAACGTGTCCAGCTCCCTAATGATC-3′. *Rbbp7* sgRNA sequences are 5′-CACCGCCCAGCACTAGCCAATGAA-3′ and 5′-AAACTTCATTGGCTAGTGCTGGGC-3′. The modification of *Rbbp4/7* genes in ESCs followed the procedure as described (Yang *et al.* 2014). Briefly, 5 × 10^4^ E14 ESCs were cultured on 60 mm dishes for 1 day and then transfected with plasmids expressing Cas9 and sgRNAs, along with a plasmid expressing PGK-PuroR (Addgene, Cat. No. 31937) using the FuGENE HD reagent (Promega) according to the manufacturer’s instructions. The cells were treated with 2 μg/ml puromycin for 2 days and recovered in a standard culture medium until ESC colonies grew. Targeted colonies were genotyped by PCR and verified by DNA sequencing and Western-blot analyses.

### ChIP-seq and ChIP-PCR analyses

ChIP was performed as described (Raab *et al.* 2015) with minor modifications. Five million cells were used per IP. The nuclear membrane was broken by mild sonication instead of passing through 20G needles. The chromatin was immunoprecipitated with the antibodies listed in Reagent Table. ChIP-seq libraries were prepared using the KAPA HyperPrep kit according to the manufacturer’s instructions and sequenced on Illumina’s NovaSeq 6000 system. Sequence reads were aligned to genomic sequence (mm10) with Bowtie2 (Langmead and Salzberg 2012). MACSv2 identified ChIP-seq enrichment using the broad peaks model (Zhang *et al.* 2008). Differential binding analyses were performed using CSAW, and significant differences in counts were called at a false discovery rate (FDR)≤0.05 (Lun and Smyth 2016). ChIP-qPCR validated differential binding on target loci (Supplementary Table 1) using SsoFast EvaGreen supermix (Bio-Rad) and CFX96 thermocycler (Bio-Rad).

### RNA-seq analysis

RNA-seq analysis was performed in triplicate for control and each mutant cell line. Cells were lysed with the TRIzol reagent (Invitrogen), and total RNA was isolated using the Direct-zol RNA kit (Zymo). Sequencing libraries were prepared using a Kapa mRNA HyperPrep kit per the manufacturer’s instructions and then sequenced on an Illumina NovaSeq 6000 system (paired-end×50 bp). Sequence reads were aligned to the mm10 genomic sequence with STAR (v2.7.3) (Dobin *et al.* 2013). Aligned reads were counted by HTSeq (Anders *et al.* 2005), and differentially expressed genes between controls and mutants were analyzed using DESeq2 (v1.22.2). Significant differences in counts were called at FDR adjusted *P*-values ≤0.01 (Love *et al.* 2014).

### Protein extraction, histone extraction, immunoprecipitation, and Western blotting

Protein or histones were prepared from ESCs for Western-blot analysis as described (Mu *et al.* 2017, p. 1). For immunoprecipitation assays, whole-cell lysate was prepared in F lysis buffer (20 mM Tris pH 7.9, 500 mM NaCl, 4 mM MgCl_2_, 0.4 mM EDTA, 2 mM DTT, 20% glycerol, 0.1% NP40, and proteinase inhibitor) and adjusted to 300 mM NaCl by adding dilution buffer (20 mM Tris pH 7.9, 10% glycerol) (Cao and Zhang 2004, p. 12). Protein extract (200 μg) was incubated with antibodies (see Reagents Table) for immunoprecipitation or Western blot.

## Results

### Generation of stable *Rbbp4* and *Rbbp7* mutant ESC lines by CRISPR

**Fig. 1.**
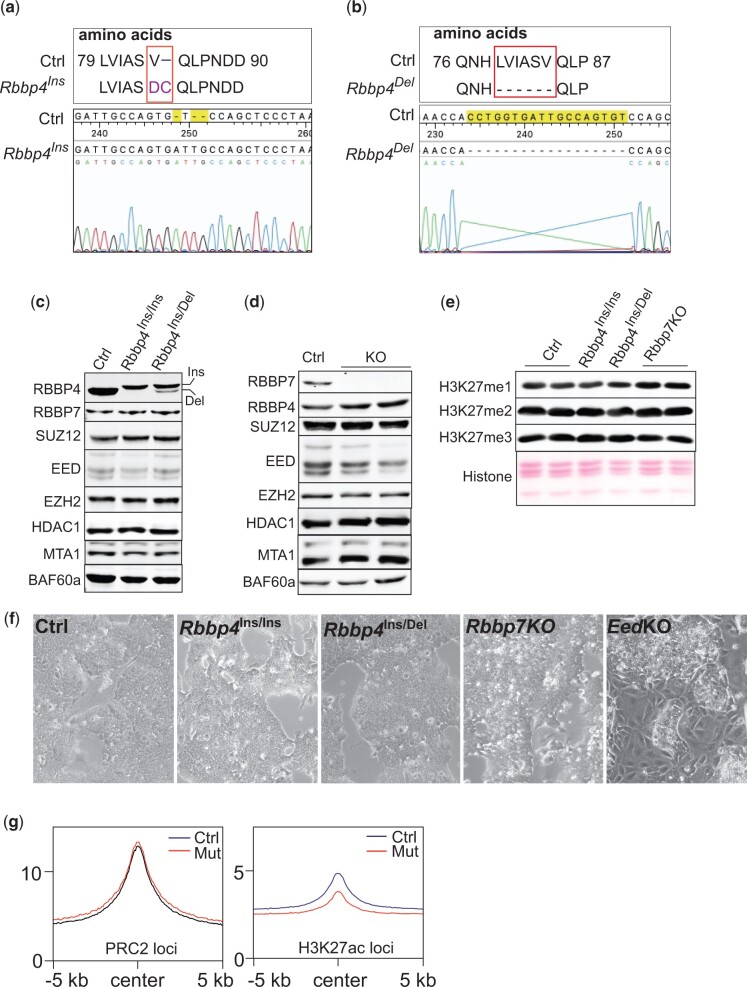
Characterization of *Rbbp4* and *Rbbp7* mutant ESC lines generated by CRISPR-Cas9. a) Identification of a 3-nucleotide insertion in *Rbbp4*^Ins/Ins^ ESC line by DNA sequencing caused amino acid substitution and insertion in mutant RBBP4. b) Identification of an 18-nucleotide deletion in the *Rbbp4*^Ins/Del^ ESC line by DNA sequencing caused 6-amino acid truncation in the mutant RBBP4. c) Assessment of RBBP4 and other subunits of RBBP4-containing complexes in E14 (ctrl) and 2 *Rbbp4* mutants ESC lines by Western-blot analysis. BAF60a serves as a loading control. d) Assessment of RBBP7 and other subunits of RBBP7-containing complexes in E14 (ctrl) and *Rbbp7* knockout (KO) ESCs by Western-blot analysis. e) Western-blot analysis of H3K27 methylation in E14 (ctrl), *Rbbp4* mutant, and *Rbbp7* knockout (KO) ESCs. f) Comparative brightfield microscopy images of ESC colonies in culture. g) Profile plots depicting the enrichment of RBBP4 within −5/+5 kb of the center of SUZ12 binding regions and K27ac enriched regions.

To generate viable cell lines with disrupted RBBP4 or RBBP7 function, we utilized CRISPR-Cas9 to target the N-terminus of RBBP4 and RBBP7 with sgRNAs to isolate viable mutant ESC clones. Mutant cell lines were validated by examining nucleotide sequences and protein expression levels of *Rbbp4* and *Rbbp7*. For *Rbbp4*, we obtained 2 ESC clones. The first mutant clone, *Rbbp4^Ins/Ins^*, contains a 3-nucleotide insertion on both alleles, resulting in a homozygous missense mutation (Val84Asp) and a Cys insertion at the 85th amino acid (Fig. 1a). The second mutant clone, *Rbbp4^Ins/Del^*, has the same *Rbbp4^Ins^* on 1 allele and an 18-nucleotide deletion on the other allele, leading to the deletion of 6 amino acids (79th to 84th) (Fig. 1b).

Western-blot analysis showed that mutant RBBP4 proteins expressed at lower levels and RBBP4^Ins^ migrated slower relative to controls (Fig. 1c). The gel shift associated with RBBP4^Ins^ could be due to oxidation of the inserted cysteine, which can retard protein migration through SDS–PAGE (Wu *et al.* 2000). We did not retrieve *Rbbp4* homozygous null clones confirming *Rbbp4* as an essential gene (Huang *et al.* 2021, p. 4). For *Rbbp7*, we obtained 2 homozygous null ESC clones with no RBBP7 protein detected (Fig. 1d), suggesting RBBP7 is dispensable for ESC viability. The mutations in *Rbbp4* or loss of RBBP7 did not impair the expression of PRC2 core subunits, EED, EZH2, and SUZ12, and HDAC complex subunits such as MTA1 and HDAC1 (Fig. 1, c and d). Global levels of H3K27 methylation were not altered in *Rbbp4* and *Rbbp7* mutants as assessed by Western blot (Fig. 1e). Both *Rbbp4* mutants and *Rbbp7* knockout cells maintained their self-renewal capacity and did not exhibit spontaneous differentiation that occurred in *Eed* knockout ESCs (Fig. 1f).

To test whether the *Rbbp4* mutations impaired its binding to chromatin, we performed ChIP-seq analyses to determine RBBP4 enrichment on PRC2-targeted loci and H3K27ac enriched regions. Although RBBP4 protein was lower in the mutant cells, there was no reduction of mutant RBBP4 on PRC2 target loci indicated by SUZ12 binding regions (Fig. 1g). In contrast, the decrease observed in mutant RBBP4 peaks at H3K27ac enriched regions (Fig. 1g) results from either insufficient RBBP4 or impaired binding capability of mutant RBBP4 to chromatin.

### RBBP4 is required for PRC2 assembly and trimethylation of H3K27 on target chromatin

**Fig. 2.**
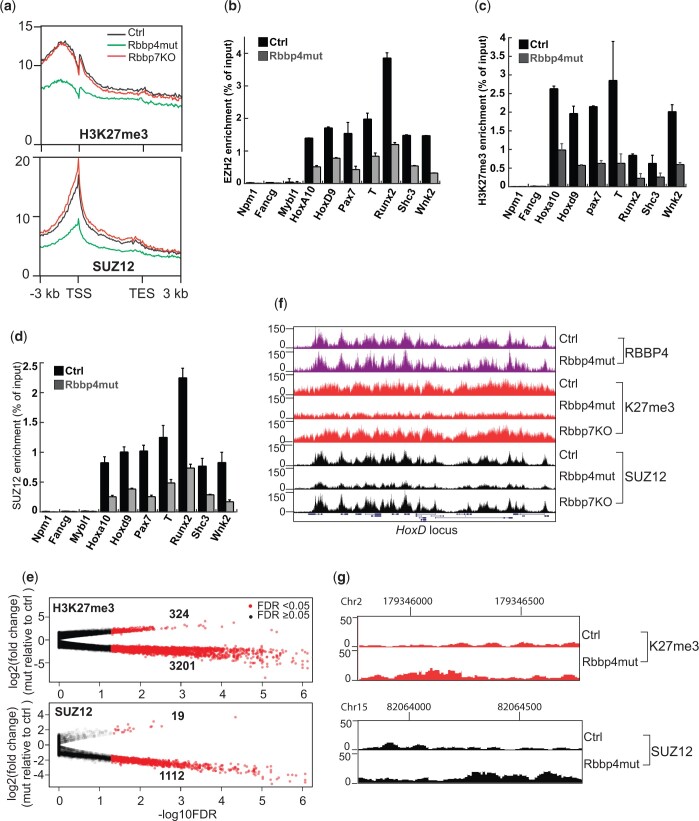
Rbbp4 mutations impaired PRC2 assembly and trimethylation of H3K27 on target chromatin. a) Profile plots scoring overall enrichment of H3K27me3 and SUZ12 across gene bodies in the genome. TSS, transcription start site; TES, transcription end site. ChIP-seq data from the 2 *Rbbp4* mutants were combined and labeled as Rbbp4mut since there was no difference between these 2 mutants. b) ChIP-PCR analysis of EZH2 enrichment on PRC2 target genes. c) ChIP-PCR analysis of H3K27me3 enrichment on PRC2 target genes. *Npm1*, *Fancg*, and *Mybl1* serve as negative controls not targeted by PRC2. d) ChIP-PCR analysis of SUZ12 enrichment on PRC2 target genes. e) Comparison of H3K27me3 and SUZ12 enrichment on binding loci between controls and *Rbbp4* mutants. Each dot represents a genomic region with H3K27me3 or SUZ12 binding. f) Genome browser images of the *HoxD* locus marked by H3K27me3, RBBP4, and SUZ12. g) Genome browser image of a genomic region with increased SUZ12 and H3K27me3 in *Rbbp4* mutant ESCs by differential binding analysis.

We performed ChIP-seq analyses to examine H3K27me3 enrichment and SUZ12 binding on PRC2 target loci. Although global levels remained stable (Fig. 1e), reduced H3K27me3 enrichment across gene bodies occurred in *Rbbp4* mutants compared to the controls (Fig. 2a). A decrease in SUZ12 and EZH2 binding at target genes accompanied the reduction in H3K27me3 (Fig. 2, a and b). We confirmed ChIP-seq results by ChIP-qPCR analysis on genes with H3K27me3 localized broadly across gene bodies (*Hoxa10*, *Hoxd9*, *Pax7*, *T*) or narrowly around the TSS (*Runx2*, *Shc3*, *Wnk2*) (Fig. 2, c and d). In contrast, the loss of RBBP7 did not reduce the enrichment of H3K27me3 and SUZ12 on PRC2 loci (Fig. 2a). Together these data suggest that RBBP4 is essential for PRC2 binding on target chromatin to establish H3K27me3.

Since the reduction of H3K27me3 on PRC2 loci did not affect global levels of H3K27me3 in mutant cells (Figs. 1e and 2a), we asked whether RBBP4 dysfunction led to a redistribution of PRC2 and H3K27me3 across the genome. Differential binding analysis revealed 3,201 and 1,112 genomic loci with decreased H3K27me3 and SUZ12, respectively (Fig. 2e). Most loci with depleted H3K27me3 are essential developmental genes such as the Hox gene clusters (Fig. 2f). Fewer genomic regions with increased SUZ12 and H3K27me3 occupancy occurred in RBBP4 mutants (Fig. 2e). These sites displayed narrow SUZ12 and H3K27me3 enrichment that failed to meet the threshold of the commonly used peak caller, MACS2 (Fig. 2g). Truncated PRC2 that retains catalytic activity but loses binding capability can maintain global levels of H3K27me3 at lower levels in non-PRC2 target regions not detectable by ChIP-seq (Højfeldt *et al.* 2018). These data might explain the patterns of SUZ12 and H3K27me3 in RBBP4 mutant cells. Nevertheless, our findings indicate that RBBP4 plays an essential role in effective PRC2 binding to target chromatin.

### RBBP4 disruption reshapes the genomic landscape of H3K27ac

**Fig. 3.**
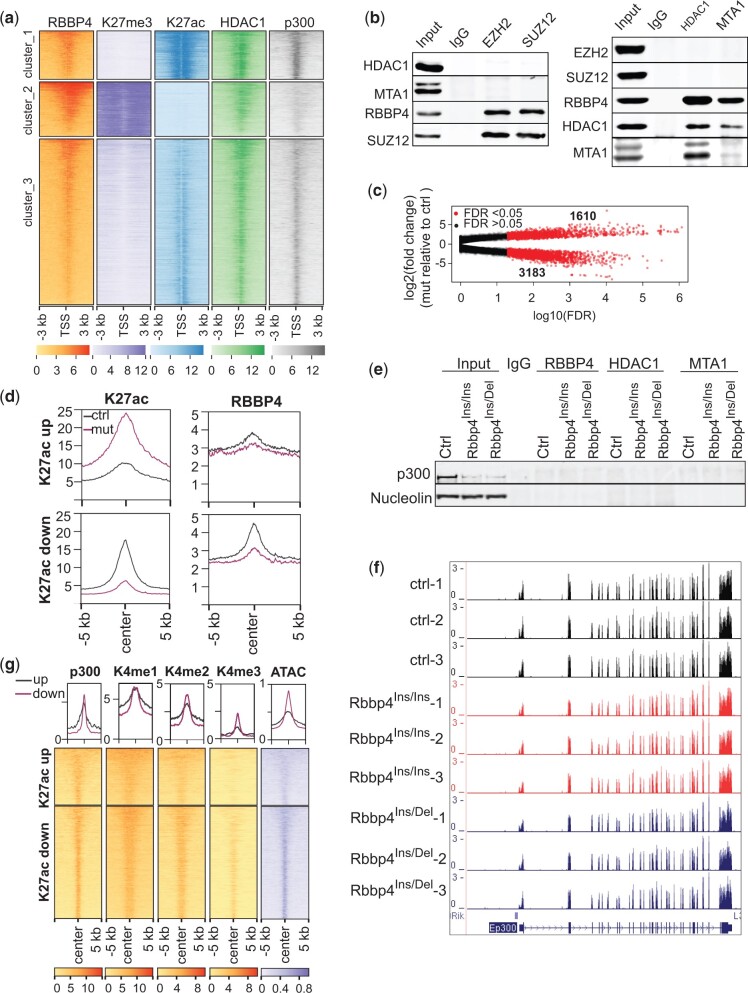
RBBP4 controls acetylation levels of H3K27 on its target chromatin. a) Clustering RBBP4 binding sites based on methylation and acetylation status of H3K27 within −3/+3 kb of the transcription start sites. b) Coimmunoprecipitation and Western-blot analysis showed no interaction between PRC2 and HDAC1-containing complex. c) Comparison of H3K27ac enrichment on its binding loci between controls and *Rbbp4* mutants. Each dot represents a genomic region with H3K27ac binding. d) Profile plots depicting the enrichment of RBBP4 within ±5 kb of the center of deregulated H3K27ac loci in *Rbbp4* mutants. e) Coimmunoprecipitation and Western-blot analysis on the interaction between p300 and the subunits of HDAC-containing complexes. f) UCSC genome browser snapshot of p300 transcriptional levels by RNA-Seq analysis. g) Heatmaps examining the genomic regions which had altered H3K27ac in Rbbp4 mutants for marks of enhancer signatures.

Since RBBP4 exists in chromatin-modifying complexes responsible for methylation and deacetylation of H3K27, we clustered binding loci into 3 groups based on the enrichment patterns of H3K27me3, H3K27ac, HDAC1, and p300 (Fig. 3a). Cluster_2 represents PRC2 targeted loci as indicated by strong H3K27me3 signals. This cluster also exhibited enrichment of HDAC1 with concomitant depletion of H3K27ac. However, PRC2 and HDAC1-containing complexes did not interact with each other (Fig. 3b). We postulated that HDAC1 prevented the assembly of activating histone acetylation complexes in these regions, while H3K27 residues are methylated to maintain a repressive chromatin state. The other 2 RBBP4 clusters were marked by high and medium levels of H3K27ac and p300, respectively, even though enriched for HDAC1 (Fig. 3a). In interphase nuclei, acetylation and deacetylation occur dynamically at specific sites and within large regions in chromatin, which delineates the formation of euchromatin and heterochromatin domains (Koprinarova *et al.* 2016). The colocalization of HDAC1 and p300 suggests that these genomic regions experience dynamic changes in H3K27ac levels.

Since RBBP4 is the core component of several histone deacetylase complexes(Kelly and Cowley 2013), disruption of RBBP4 would result in an increase of H3K27ac on chromatin. Differential binding analysis of H3K27ac in E14 and *Rbbp4* mutant ESCs did reveal numerous loci (1,610) with increased H3K27ac. However, to our surprise, there were approximately 2-fold more (3,183) genomic regions with decreased H3K27ac levels (Fig. 3c). In mutants, RBBP4 binding was reduced at loci associated with increased (K27ac up) or reduced (K27ac down) levels of K27ac (Fig. 3d), suggesting the involvement of RBBP4 in regulating H3K27 acetylation levels.

We found protein levels of p300, a histone acetylase specialized for H3K27ac, were strikingly decreased in the mutants (Fig. 3e), which may explain the extensive reduction of H3K27ac levels in mutant cells. However, p300 mRNA levels were not reduced in *Rbbp4* mutants compared to the control (Fig. 3f), suggesting the possible involvement of RBBP4 in stabilizing p300 protein. However, no physical interaction between p300 and RBBP4/HDAC1/MTA1 was detected (Fig. 3e). Therefore, 1 future question is how the crosstalk between H3K27ac writer and eraser complexes coordinates to regulate acetylation levels.

To characterize the chromatin signature of genomic loci with increased and decreased levels of H3K27ac in the mutants, we retrieved ChIP-seq data for H3K4me1 (GSM1908888), H3K4me2 (GSM881353), H3K4me3 (GSM723017), p300 (GSM2360934), and ATAC-seq (GSE120393) in mouse ESCs from GEO. We examined the distribution and enrichment of these features around H3K27ac regions. H3K27ac combined with H3K4 methylation, p300, and open chromatin indicated by ATAC are used to predict cell type-specific enhancers (Catarino and Stark 2018). The colocalization of altered H3K27ac loci with these enhancer marks (Fig. 3g) suggests that mutations of RBBP4 can impact enhancer activity, leading to deregulation of transcription.

### RBBP4 disruption led to transcriptional misregulation

**Fig. 4.**
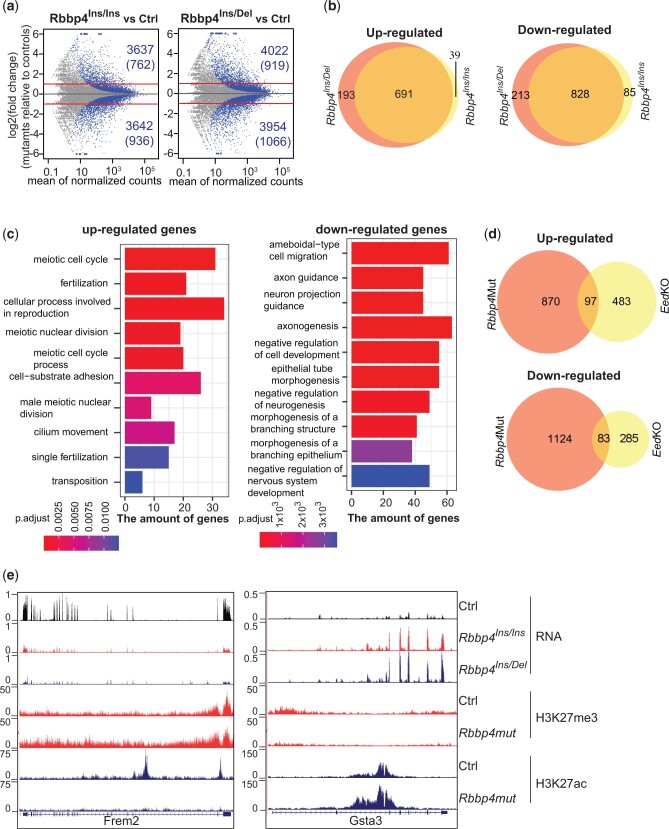
RBBP4 disruption led to transcriptional misregulation. a) MA-plot shows differential gene expression due to RBBP4 dysfunction. Colored dots represent genes with significant changes in transcription (*P*< 0.01). Lines indicate 2-fold changes. b) Venn diagrams show the overlap of differential expressed genes with more than 2-fold difference between *Rbbp4*^Ins/Ins^ and *Rbbp4*^Ins/Del^ ESCs. c) Gene ontology (GO) analysis of up- and downregulated genes shared by the 2 *Rppb4*mutants. About 712 upregulated and 851 downregulated genes were included in this analysis. The top 10 most enriched biological processes based on their *P*-values are shown. d) Venn diagrams showing the overlap of differential expressed genes with more than 2-fold change between *Rbbp4* mutant and *Eed* knockout ESCs. e) Genome browser images of representative genes, the transcription of which was inversely deregulated in *Rbbp4* mutants and *Eed* knockout.

RNA-seq analysis performed to assess gene expression changes due to RBBP4 disruption revealed that RBBP4 mutations dramatically altered transcriptional profiles. In *Rbbp4^Ins/Ins^*, 3,637 genes showed upregulated expression (*P* < 0.01), while 3,642 showed reduced expression relative to controls. Similar genes showed misregulation in *Rbbp4^Ins/Del^*, with 4,022 upregulated and 3,954 downregulated. For those differentially expressed genes with more than a 2-fold change in transcription and a mean read count of more than 20, 762, and 919 genes showed upregulation, and 936 and 1,066 showed decreased expression *Rbbp4^Ins/Ins^* and *Rbbp4^Ins/Del^*, respectively (Fig. 4a). These differentially expressed genes showed similar transcriptional patterns between the 2 mutants. They shared 691 upregulated, and 828 downregulated genes (fold change >2 and read count >20) (Fig. 4b). Gene ontology (GO) analyses indicated that the shared genes affected reproductive processes and neural development (Fig. 4c).

We compared gene expression changes due to RBBP4 depletion and loss of PRC2 function. In *Eed* knockout ESCs, 580 and 368 genes’ transcription increased and decreased by more than 2-fold, respectively (Fig. 4d) (Das *et al.* 2015), compared to the 967 and 1,207 genes with observed changes in RBBP4 mutants. There were only 97 upregulated and 83 downregulated genes shared by *Eed* knockout and *Rbbp4* mutants (Fig. 4d). Unexpectedly, 115 misregulated genes in the *Eed* knockout showed inverse regulation with dramatic changes in *Rbbp4* mutants (Supplementary Table 2). For example, the expression of *Frem2* and *Gsta3* are up- and downregulated, respectively, in *Eed* knockout ESCs but oppositely regulated in *Rbbp4* mutants (Fig. 4e and Supplementary Table 2). These data suggest that RBBP4 controls gene activity independently of PRC2-mediated transcriptional repression by regulating H3K27ac levels.

### Transcriptional misregulation in *Rbbp4* mutants was associated with altered enhancer associated H3K27 acetylation

**Fig. 5.**
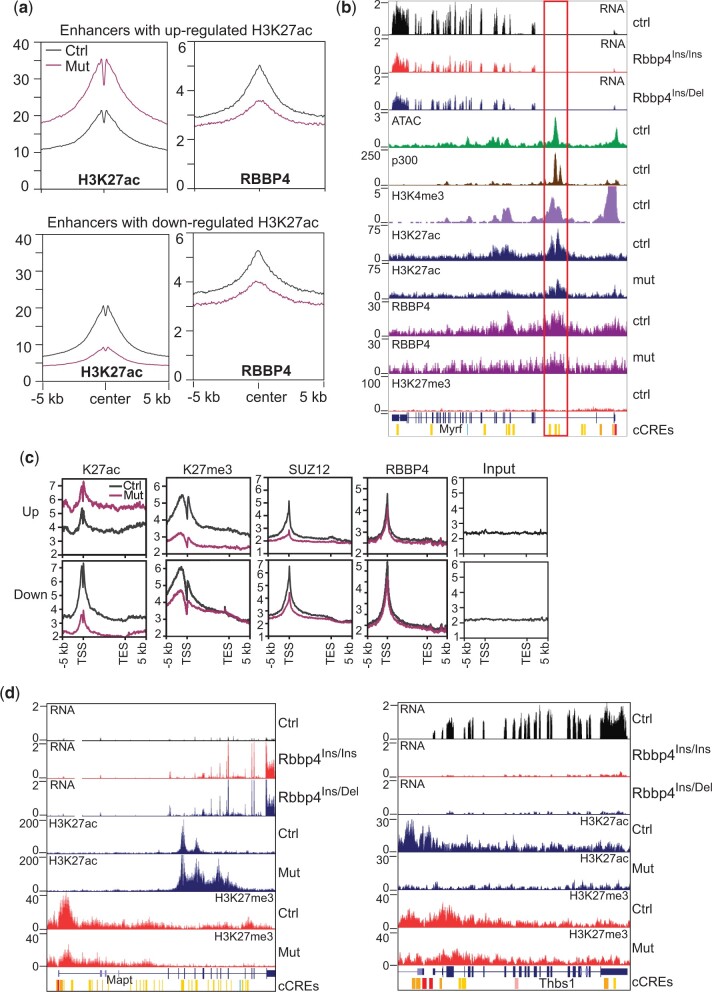
RBBP modulated H3K27ac levels on enhancers to influence gene activity. a) Profile plots depicting reduced RBBP4 binding within ±5 kb of the center of enhancers with deregulated H3K27ac in the mutant. b) Genome browser image of *Myrf* locus. Rectangle indicates the *Myrf* enhancer region. c) Comparison of the enrichment of H3K27ac, H3K27me3, RBBP4, and SUZ12 on up- and downregulated genes between controls and *Rbbp4* mutants. d) Genome browser images of representative genes whose activation and silencing are coordinated with the gain and loss of H3K27ac on cis-regulatory elements.

To explore the mechanisms by which changes in H3K27ac in response to the loss of RBBP4 perturbs gene expression, we characterized the genomic features of regions typically marked by H3K27ac. Using the ENCODE mouse cis-regulatory elements database as a reference, we found both up- and downregulated H3K27ac loci enriched with enhancer-like signatures (5,836 and 7,165, respectively) (Table 1). As defined by DNase hypersensitivity, H3K4me3, and CTCF binding, selected loci cover promoters and other cis-regulatory elements (Table 1). The enhancers with both increased and decreased H3K27ac in mutant ESCs had reduced RBBP4 binding (Fig. 5a), suggesting that RBBP4 is involved in regulating enhancer activity.

**Table 1.** The mutants’ genomic regions with altered H3K27ac are enriched with predicated cis-regulatory elements.

Genomic feature	K27ac-Up	K27ac-Down
Promoter	494	1,074
Proximal enhancer	1,621	2,694
Distal enhancer	4,215	4,471
Super-enhancer	59	17
Regions with other CRE	1,053	2,286

We examined the impact of H3K27ac and RBBP4 levels on the activity of validated enhancers. For example, in mouse oligodendrocytes, a *Myrf* intron is associated with an enhancer (Kim *et al.* 2019). This enhancer is also active in mouse ESCs as indicated by several features, including H3K27ac, H3K4me3, p300 chromatin marks, open chromatin ATAC signature, and high *Myrf* transcription levels (Fig. 5b). In *Rbbp4* mutant cells, RBBP4 binding and H3K27ac accumulation were decreased around the *Myrf* enhancer, resulting in transcriptional repression (Fig. 5b). Thus, RBBP4 can control gene activity by regulating H3K27ac levels on enhancers.

We examined the enrichment of H3K27ac, H3K27me3, RBBP4, and SUZ12 on commonly misregulated genes in the 2 *Rbbp4* mutants (Fig. 5c). In mutants, the incorporation of H3K27ac was markedly increased across upregulated genes and decreased for downregulated genes. H3K27me3 and SUZ12 levels were lower on both up- and downregulated genes because disruption of RBBP4 caused a broad reduction of H3K27me3 and SUZ12 binding. For instance, *Mapt* expression increased by 1.4-fold upon depletion of H3K27me3 in *Eed* KO ES cells (Das *et al.* 2015). In contrast, a 14.4-fold increase in *Mapt* expression coincided with elevated H3K27ac spanning predicted cis-regulatory elements accompanied by only a partial loss of H3K27me3 in *Rbbp4* mutant ESCs (Fig. 5d). Thus, enhanced activation of *Mapt* in *Rbbp4* mutants was primarily related to the acquisition of H3K27ac rather than a loss of H3K27me3. We also observed that *Thbs1* was silenced with loss of H3K27ac upstream of its promoter, but not via gaining repressive H3K27me3 (Fig. 5d). Together, these data suggest that RBBP4-mediated metabolism of H3K27ac on chromatin is involved in determining gene activity.

### RBBP4 binding on super-enhancers may regulate stemness gene expression in ESCs

**Fig. 6.**
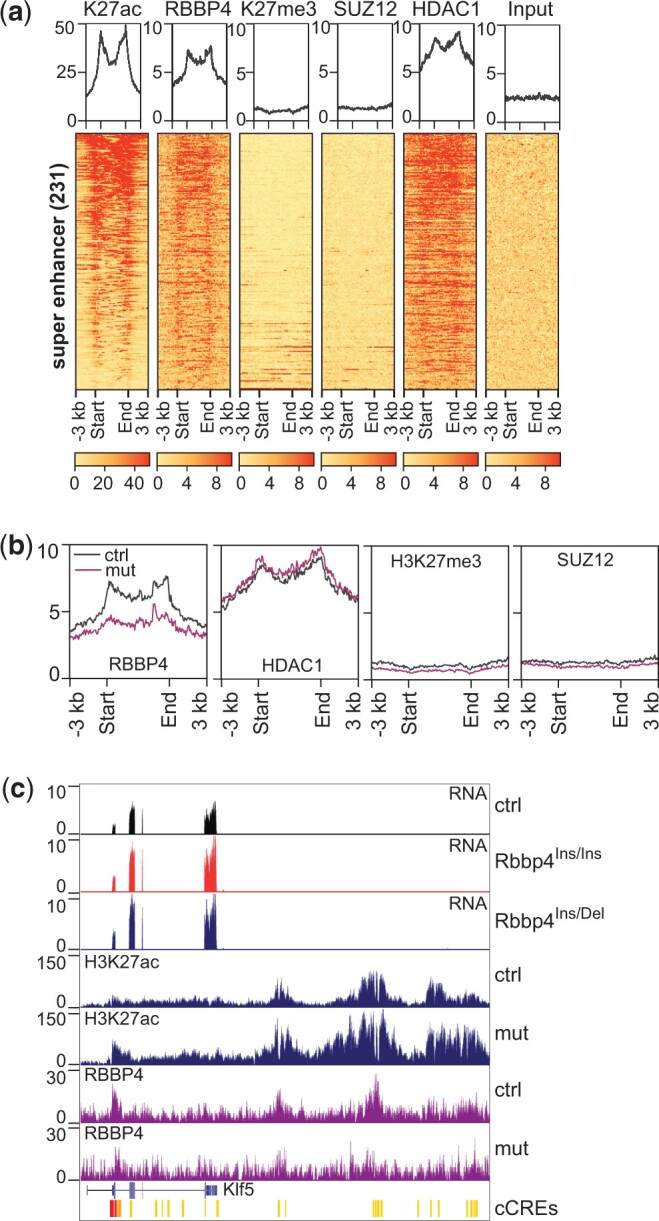
Reduced RBBP4 on super-enhancers due to *Rbbp4* mutations is related to changes in stemness gene expression in ESCs. a) RBBP4 and HDAC1 are enriched on super-enhancers. Heatmaps cover the regions within 3 kb upstream and downstream of super-enhancers. b) Profile plots depicting the enrichment of RBBP4, HDAC1, H3K27me3, and SUZ12 within 3 kb upstream and downstream of super-enhancers. c) Genome browser images showing increased H3K27ac on a putative *Klf5* enhancer in *Rbbp4* mutants in relation to the upregulation of this gene.

Super-enhancers are marked by clusters of H3K27ac and function to maintain high expression of cell-type-specific genes for cell identity maintenance (Hnisz *et al.* 2013). We found dysregulation of H3K27ac through abnormal enrichment on super-enhancers in *Rbbp4* mutant ESCs. Among the 231 super-enhancers present in mouse ESCs (Khan and Zhang 2016), 76 super-enhancers underwent H3K27ac changes in RBBP4 mutants (Table 1). RBBP4 and HDAC1 bind to super-enhancer regions and exhibit similar distribution patterns as H3K27ac (Fig. 6a). Compared to E14 cells, less RBBP4 is associated with super-enhancers in *Rbbp4* mutants, but this did not affect the binding of HDAC1 at these regions (Fig. 6b).

We next explored individual contexts of relevant gene modulation in ESCs as RBBP4 is involved in maintaining mouse ESC pluripotency (Huang *et al.* 2021, p. 4). We focused on *Klf5* since it regulates ESC pluripotency (Parisi *et al.* 2008) and prevents differentiation toward mesoderm (Aksoy *et al.* 2014). Given that *Klf5* is necessary for controlling and defining ESC identity, we predicted that the *Klf5* locus and its upstream region, broadly marked by H3K27ac (Fig. 6c), is a super-enhancer. Here, we observed elevated H3K27ac levels across this region along with decreased binding of RBBP4 in mutant cell lines (Fig. 6c). Correspondingly, the transcription of *Klf5* was enhanced (Fig. 6c). Given these specific examples and genome-wide data, our analysis indicates that RBBP4 adjusts H3K27ac levels at super-enhancers to finely tune gene expression as necessary for stem cell homeostasis and differentiation.

## Discussion

As a core subunit of PRC2, RBBP4 is a histone chaperone protein that directly binds to histones H3 and H4 (Verreault *et al.* 1996). It is also a core component of multiple chromatin-modifying complexes. It is essential for viability, making it difficult to study specific roles in PRC2-related histone methylation vs HDAC-related acetylation. Using the CRISPR-Cas9 system to introduce mutations in *Rbbp4*, we generated viable ESC lines with an expression of altered RBBP4 proteins. These mutations resulted in striking changes in the genomic enrichment and distribution of both H3K27me3 and H3K27ac, as well as a reshaped transcriptional profile.

Regarding PRC2, disruption of RBBP4 impairs the recruitment of SUZ12 and EZH2 to PRC2 target loci, leading to a decrease in H3K27me3. A recent study demonstrated that SUZ12 binds to PRC2 target regions independently of EED and EZH1/2, which is essential for guiding PRC2 to its correct genomic loci (Højfeldt *et al.* 2018). We found that RBBP4 disruption did not affect protein levels of PRC2 subunits but interfered with SUZ12 recruitment to PRC2 loci, suggesting that RBBP4 association with chromatin is also an early event in PRC2’s assembly on target genomic regions. SUZ12–RBBP4 complexes also guide the incorporation of ancillary subunits to form different PRC2 subcomplexes on chromatin (Chen *et al.* 2018, 2020). It is possible that RBBP4 and SUZ12 coordinate to initiate PRC2 assembly on specific genomic sites.

Compared to a developmental arrest at the gastrulation stage due to depletion of EED (Faust *et al.* 1998), SUZ12 (Pasini *et al.* 2004, p. 12), and EZH2 (O’Carroll *et al.* 2001, p. 2), RBBP4 ablation resulted in more severe phenotypes such as preimplantation lethality and failure of inner cell mass outgrowth (ICM) (Miao *et al.* 2020, p. 4). These data indicate that non-PRC2-related functions such as RBBP4-mediated histone deacetylation are crucial for ICM proliferation and lineage commitment. RBBP4 coexists with HDAC1 in deacetylase complexes that broadly deacetylatelysines (Johnson *et al.* 2002). One of these, the NuRD complex, where RBBP4, MTA1, and HDAC1 are core subunits, was found to bind to nearly all active enhancers and promoters in ESCs (Hoffmann and Spengler 2019). Based on these results and the knowledge that H3K27ac is enriched in cis-regulatory elements to regulate gene activity, in this study, we also focused on the impact of RBBP4 on acetylation status. As expected, RBBP4 disruption caused extensive alteration of H3K27ac on enhancers, further indicating the importance of RBBP4-containing complexes in gene activation states.

Since RBBP4 both methylates and deacetylates H3K27, we investigated their roles in transcriptional regulation. Our data showed that genomic distribution of these 2 modifications is mutually exclusive, and physical interaction between PRC2 and HDAC-containing complexes was not detected, indicating that methylation and deacetylation of H3K27 are largely independent events in gene repression. In support of this, we found relatively few overlapping dysregulated genes between *Rbbp4* disruption and *Eed* knockout in ESCs. Compared to the ablation of EED, SUZ12, and EZH2, RBBP4 disruption caused more widespread transcriptional deregulation (Pasini *et al.* 2007; Das *et al.* 2015; Huang *et al.* 2021). We also found a subset of genes discordantly regulated between *Eed* and *Rbbp4* mutants concerning loss or gain of H3K27ac. Together, these data suggest that RBBP4 controls gene activity primarily through regulating H3K27ac levels.

A previous study showed sparse increases of H3K27ac throughout the genome as a consequence of PRC2 loss and suggested chromatin hyperacetylation rather than specific loss of repressive control at target genes leads to the early developmental failure induced by PRC2 disruption (Lavarone *et al.* 2019). In mouse ESCs, many *Hox* cluster genes enriched with H3K27me3 did not experience transcriptional activation due to the loss of H3K27me3 (Pasini *et al.* 2007; Chamberlain *et al.* 2008). H3K27me3 marks poised chromatin maintaining a transcriptionally silenced state. This silencing is critical for spatiotemporal activation of cell-type-specific genes during development. In contrast, H3K27ac is a more potent gene expression activator that dynamically regulates transcriptional levels.

In summary, our *Rbbp4* mutant cellular models demonstrated crucial roles of RBBP4 in H3K27 methylation and acetylation. RBBP4 is required to recruit SUZ12 to PRC2 target loci, indicating RBBP4 is a determining factor for site-specific H3K27me3 across the genome. In contrast, RBBP4 facilitates the deacetylase activity of HDAC complexes for the efficient removal of the acetyl group on H3K27. Meanwhile, RBBP4 also promotes H3K27ac by maintaining p300 levels. Altogether, therefore, it is possible that RBBP4 assists in controlling H3K27ac levels on cis-regulatory elements for exquisite programming of the transcriptome with regards to cellular context. Understanding the molecular mechanisms of RBBP4 will elucidate its functional specificities in chromatin regulation and gene expression.

## Data availability

ChIP-seq and RNA-seq data are accessible at the Gene Expression Omnibus database repository GSE183291 (RNA-seq) and GSE183292 (ChIP-seq).

Supplemental material is available at *G3* online.

## Supplementary Material

jkac082_Supplementary_TableS1

jkac082_Supplementary_TableS2
