# Supplementary material for: RBBP4 dysfunction reshapes the genomic landscape of H3K27 methylation and acetylation and disrupts gene expression
Source: G3 (Bethesda). 2022 Apr 13;12(6):jkac082. doi: 10.1093/g3journal/jkac082 (PMC9157164; doi:10.1093/g3journal/jkac082)
Supplement: jkac082_Supplementary_TableS1 [file jkac082_supplementary_tables1.pdf]

Supplementary Table S1. Primer sets for ChIP-qPCR

Gene name	Forward primers	Reverse primers
Npm1	GCGAGGCCACAAAGAGAA	TTCATCCTCACGCCTAGGTA
Fancg	TGGGCGTTCTCCACAGTACTACAA	TAGAACCAACGGCAGTCCAGGTTT
Mybl1	TATGCGGTACTTGAAGGATGGCGA	AGCAGGAAAGGACAGAGAGCAAGA
Hoxa10	CTGGCTCTTGAACCTGTACCCC	CAAGGGTGCTTCCAAATAGTC
Hoxd9	GGATAATCGCCTAGGTGTGACTTAG	CATCTCTTCTTGCCTCTCTGGG
Pax7	AGCAGAAAGAGGCGCTGAGAG	CTGAGCCCAGAGGTTGCG
T	GGGACCCAGGTGTAATCTTTG	CAACAGCCACCTTCACTTCT
Runx2	CACGACAACCGCACCAT	CACGGAGCACAGGAAGTT
Shc3	CATGAGTGCCACCAGGAAG	GTTACCCACCTTGACCACATAG
Wnk2	CTCCCGAGCCCTTCTGTA	CCTGTCTCTAGCTGTCCCA
