# Supplementary material for: RBBP4 dysfunction reshapes the genomic landscape of H3K27 methylation and acetylation and disrupts gene expression
Source: G3 (Bethesda). 2022 Apr 13;12(6):jkac082. doi: 10.1093/g3journal/jkac082 (PMC9157164; doi:10.1093/g3journal/jkac082)
Supplement: jkac082_Supplementary_TableS2 [file jkac082_supplementary_tables2.pdf]

Supplementary Table S2. Genes are inversly mis-regulated in Eed knockout and Rbbp4 mutant ESCs

gene	log2FoldChange(Rbbp4mut/wt)	log2FoldChange(EedKO/wt)
Alx1	-1.9558	2.6442
Amot	-1.9957	1.2056
Apob	-1.5935	1.9870
Ar	-3.1382	1.5437
Asxl3	-1.5608	1.4652
AU015836	-1.2552	1.9815
C130071C03Rik	-1.5742	2.5379
Calcr	-3.3209	1.1766
Car4	-3.0104	1.1960
Ccdc158	-1.1747	1.0501
Ccnd2	-4.0106	1.0568
Cdcp1	-1.0722	1.1378
Cdkn3	-1.7911	1.0976
Celf2	-1.0693	1.3820
Cftr	-3.8239	2.1674
Cldn3	-1.5230	1.0639
Clic5	-3.5270	1.1206
Col2a1	-1.3397	1.2339
Dgkb	-1.8250	1.8667
Ebf1	-1.7679	2.0986
Emilin3	-1.4707	1.6214
Enpp2	-4.4128	1.2985
Epha8	-1.5007	1.1271
Fam83b	-1.1645	1.5601
Frem2	-2.5233	1.5560
Frk	-1.7749	1.6118
Gabra4	-1.6917	2.1148
Gata4	-1.5643	2.8706
Gfra3	-3.7351	2.3475
Grm6	-2.7326	1.9359
Hoxa1	-2.5309	1.4204
Igdcc3	-1.5143	1.1019
Igf2	-1.2205	2.2124
Insm1	-3.6690	1.3266
Irs4	-2.3208	1.2046
Irx1	-4.2771	2.4901
Kctd12b	-3.4429	1.4933
Lrrn1	-1.5326	1.5284
Mecom	-2.6065	3.0897
Meis1	-2.3025	2.7610
Meis2	-3.3301	2.4409
Mmp9	-1.9654	1.4912
Mpped1	-1.8588	1.3338
Mrc1	-2.4033	1.8229
Nrk	-1.1132	1.8546
Olig3	-2.8370	2.9618
Onecut2	-1.7784	1.2278
Otop1	-3.6294	1.7451
Pax3	-4.0160	2.7308
Pde4b	-2.3421	1.6363

Pitx2	-2.2957	1.7863
Podxl	-1.4796	1.3331
Prmt8	-2.9054	1.7193
Prox1	-2.0920	1.6445
Rbm46	-1.4534	1.8666
Reln	-1.5211	1.8467
S100a13	-1.2863	1.0309
S1pr1	-2.1101	1.2260
S1pr3	-1.3197	1.1487
Scara3	-2.6369	1.6319
Sema5a	-2.7866	2.3229
She	-1.4154	1.3646
Slc30a4	-1.0605	1.0973
Slc40a1	-1.7170	1.0287
Slc8a1	-1.7458	1.8935
Sox21	-1.8188	2.6598
Spag16	-1.1779	1.3396
Stk32a	-1.6366	1.4961
Syt13	-1.9572	1.4676
Tal1	-1.5071	1.3788
Tbx15	-1.5170	3.6119
Tbx20	-2.8073	1.6996
Tbx4	-1.7083	1.2894
Thbd	-3.5523	2.5154
Tmprss2	-2.0336	1.2264
Tshz1	-2.2681	1.6924
Twist1	-1.1761	1.4405
Zadh2	-1.2405	1.3539
4933427D06Rik	3.9358	-2.4764
AI662270	2.8422	-2.3185
Apod	2.9604	-2.0969
Arhgap30	1.5387	-2.3138
Calb2	1.9003	-2.8534
Ccdc113	2.5422	-1.5163
Cpe	1.2411	-1.6111
Cpn1	1.2052	-1.0846
D630023F18Rik	1.0835	-1.1809
Ddc	2.0320	-1.0234
Dglucy	3.9191	-1.7393
Fcgr2b	1.7925	-2.3869
Fcgrt	1.7023	-1.3042
Gm19705	1.2744	-1.5641
Gm5091	1.2245	-3.4413
Gm973	1.9368	-1.4661
Gsta3	2.4942	-1.3449
Hmcn2	2.3199	-1.4874
Hnf4a	3.7219	-4.4351
Igsf23	1.1162	-2.2404
Inhbb	1.1199	-1.5406
Kdm5d	1.9114	-1.9016
Ldhc	1.2143	-2.1659
Lrmp	1.1001	-1.3888
Nkx6-3	2.2452	-3.0849
Nxf3	2.3906	-2.0523
Pnma5	1.4212	-1.2200
Prss42	1.3042	-1.9585

Ptpn7	1.3015	-1.7006
Slc47a1	1.8399	-1.3321
Tmprss5	1.1563	-1.8426
Ushbp1	3.1916	-2.8026
Usp26	2.0745	-4.2724
Wdr20rt	1.5269	-1.8604
Xlr4a	5.3950	-3.4793
Zmat4	1.4276	-1.5604
Zscan5b	2.7044	-1.8845
